# A Waist-Worn Inertial Measurement Unit for Long-Term Monitoring of Parkinson’s Disease Patients

**DOI:** 10.3390/s17040827

**Published:** 2017-04-11

**Authors:** Daniel Rodríguez-Martín, Carlos Pérez-López, Albert Samà, Andreu Català, Joan Manuel Moreno Arostegui, Joan Cabestany, Berta Mestre, Sheila Alcaine, Anna Prats, María de la Cruz Crespo, Àngels Bayés

**Affiliations:** 1 Technical Research Centre for Dependency Care and Autonomous Living—CETPD, Universitat Politècnica de Catalunya—BarcelonaTech, Rambla de l’Exposició 59-69, Vilanova i la Geltrú, 08800 Barcelona, Spain; carlos.perez-lopez@upc.edu (C.P.-L.); albert.sama@upc.edu (A.S.); andreu.catala@upc.edu (A.C.); joan.manuel.moreno@upc.edu (J.M.M.A.); joan.cabestany@upc.edu (J.C.); 2 Unidad de Parkinson y Trastornos del Movimiento (UParkinson), Passeig Bonanova 26, 08022 Barcelona, Spain; bertam8@gmail.com (B.M.); alcaine.fisioterapeuta@gmail.com (S.A.); apratsparis@gmail.com (A.P.); mcruzcrespo@gmail.com (M.d.l.C.C.); 11741abr@comb.cat (À.B.)

**Keywords:** inertial measurement unit, Parkinson’s disease, monitoring, inertial data capture, algorithm

## Abstract

Inertial measurement units (IMUs) are devices used, among other fields, in health applications, since they are light, small and effective. More concretely, IMUs have been demonstrated to be useful in the monitoring of motor symptoms of Parkinson’s disease (PD). In this sense, most of previous works have attempted to assess PD symptoms in controlled environments or short tests. This paper presents the design of an IMU, called 9 × 3, that aims to assess PD symptoms, enabling the possibility to perform a map of patients’ symptoms at their homes during long periods. The device is able to acquire and store raw inertial data for artificial intelligence algorithmic training purposes. Furthermore, the presented IMU enables the real-time execution of the developed and embedded learning models. Results show the great flexibility of the 9 × 3, storing inertial information and algorithm outputs, sending messages to external devices and being able to detect freezing of gait and bradykinetic gait. Results obtained in 12 patients exhibit a sensitivity and specificity over 80%. Additionally, the system enables working 23 days (at waking hours) with a 1200 mAh battery and a sampling rate of 50 Hz, opening up the possibility to be used for other applications like wellbeing and sports.

## 1. Introduction

Human movement monitoring with inertial measurement units (IMU) has become a field of great interest in the past few years. For example, in the health field, IMUs are used in the study of falls as a tool for detecting them [1,2]; they have been employed in the assessment of movement disorders, such as those experienced by the persons with Parkinson’s Disease (PD) [3], and to quantify and analyse movements in patients with Alzheimer’s Disease [4,5]. IMUs have also been used in patients suffering stroke [6,7] and amyotrophic lateral sclerosis rehabilitation [8].

Wearable IMUs are composed of inertial sensors based on Micro-Electro-Mechanized-Systems (MEMS) technology [9]. Moreover, these systems also include microcontrollers for computing and control purposes and other peripherals such as external memories or communication modules that permit sending information to an external device. IMUs sensors present typically a very low energy consumption, which opens up the possibility to obtain devices with long battery life. In addition, they are comfortable to be worn due to their small size. In this sense, they are portable and enable ambulatory monitoring, in contrast to other movement measurement systems such as pressure platforms or electrogoniometers. Concerning user acceptance, IMUs are usually well admitted in regards to privacy issues, in contrast to cameras at home [10]. Furthermore, IMUs enable embedding algorithms to be executed in the included microprocesor, which can be employed to detect specific events (e.g., falls, posture transitions, steps), symptoms (e.g., tremor, dyskinesia), or behaviours (e.g., gestures, amount of activity), and to provide some useful information either to experts or to users in the form of fast interaction or actuation. The analysis of human movement with inertial systems is mainly based on accelerometers [11,12]; nonetheless, some works that use gyroscopes [13] and magnetometers [14] and, to a lesser extent, barometers [15,16].

Currently, only a small group of sensor devices are capable of storing inertial signals for analysis purposes and, in addition, execute machine learning-based algorithms. Most of these sensors are presented as development kits, requiring a wired power supply or needing an extra board for sending or storing data, making its use impractical in outpatient settings. To the best of our knowledge, and as shown in Section 2 and Section 4, only the 9 × 2 sensor meets these requirements but it only operates for one day and a half, and the volatile and non-volatile memory available to include machine learning algorithms is very limited [17]. Related sensors are reviewed in Section 2 in which several commercial and research systems are presented, distinguishing among dataloggers, open source dataloggers and specific devices to detect PD symptoms.

This paper presents an IMU, called 9 × 3, prepared to be employed in clinical studies involving PD patients. The sensor is worn with a hypoallergenic neoprene belt at the waist. The 9 × 3 unit has been designed according to the requirements set by the protocol of the study, which is part of the “Freezing in Parkinson’s Disease: Improving Quality of Life with an Automatic Control System” (MASPARK) project [18]. This project requires wearing an IMU during waking hours (approximately 10 h) during 30 days in order to analyse the motion and PD symptoms along several days. Hence, the system should be capable of capturing and storing raw inertial signals continuously during 10 h. However, since the patient can forget to charge the device, it has been considered that it should last for 2 or 3 days working continuously without charging the battery. Furthermore, the second stage of the clinical study requires sending alarms provoked by the presence of specific symptoms (freezing of gait and bradykinetic gait) to an actuation system consisting of a wireless headset. In addition, the 9 × 3 unit must record patients’ symptoms autonomously at their homes.

The following sections address the design of the IMU device, which is devoted to the long-term capturing of inertial signals and the real-time processing of the monitoring algorithms. Once designed, the device has been used in laboratory conditions and two different results are presented. First, results of the device being used by 12 PD patients are presented in terms of specificity and sensitivity in detecting FoG episodes and bradykinetic gait. Second, results on the battery lifetime of the sensor are described, regarding power consumption, autonomy and algorithmic execution time. The inertial system presented is an improved new version of the 9 × 2 device [17], in which only the enclosure has been kept. The design of the printed-circuit board, the microcontroller, the power management system and the used sensors are new, enhancing by far the characteristics of the previous system (as discussed in Section 4 and Section 7).

Hence, the contributions of this paper are threefold. First, a device for the long-term monitoring patients with movement disorders is presented. Second, it is demonstrated the capability of the device for detecting freezing of gait (FoG) episodes and bradykinetic gait in real-time with 12 PD patients. Finally, the device’s autonomy is analysed and shown to be able for long-term monitoring, being higher than the sensors with similar hardware.

The paper is organized as follows: Section 2 reviews the current inertial systems to perform data logging and to detect PD symptoms. Section 3 explains the requirements of the 9 × 3 unit regarding the ability to reliably capture raw signals and to execute machine learning algorithms in real time. Then, Section 4 and Section 5 describe the hardware and the firmware respectively. Section 6 is devoted to describing the experiments performed to test the system, and Section 7 reports the results obtained. Finally, Section 8 presents the conclusions.

## 2. Currently Existing Inertial Systems

IMUs have become the most employed devices for measuring movement in ambulatory conditions since they are small, light and portable devices. Furthermore, IMUs provide a wide variety of functions with the aim of using them in different fields. 

As the present work is focused on PD assessment, we present the currently available IMUs by dividing them into three groups: those which are used for data-logging purposes, those which permit to embed custom code in order to execute an algorithm in real-time, and those which are used for detecting PD symptoms in real-time.

### 2.1. Dataloggers

Among currently available IMU dataloggers, there exists a wide variety of systems. In this subsection, several systems are compared regarding the sampling frequency, autonomy, size, weight, storage unit availability, wireless capacity, and number and types of sensors. Several companies have released an inertial system series, each one with different features focused on varied applications. Furthermore, it is interesting to analyse those inertial systems, which also have a wireless connection in order to provide an alarm, get the current time by synchronizing with an external system or simply send raw data wirelessly.

A review of some commercial IMUs has been performed and it is summarised in Table 1 [17,19,20,21,22,23,24,25,26,27,28,29,30,31,32]. The main features compared are the size, weight, number and types of sensors, sampling frequency and some information about the autonomy of the system such as the consumption or battery life/capacity. In the latter feature, manufacturers do not provide all the information. Finally, the previous version of the IMU presented in this paper (formally 9 × 2) is also included in this table for comparison purposes.

In general, due to recent technological advances, electronic devices have improved with respect to previous IMUs presented in 2013 [17]. More concretely, there are three features that have been significantly improved. Firstly, the sampling frequency is higher, achieving sampling frequencies up to 1000 Hz in some IMUs, compared to 200 Hz achieved by few IMUs in 2013. Secondly, inertial systems currently tend to incorporate new sensors such as MEMS-based barometers. Barometers have been shown to be suitable sensors for contextualizing activities that involve a change in altitude such as human falls [16,33]. Thirdly, the autonomy, improving from few hours to several days (e.g., 9 × 2 lasted 36.8 h and now it lasts up to 12 days with the same battery capacity). Size and weight are features that remain static since they depend on the battery size and, in this sense, there have not been notable improvements.

### 2.2. Open Source Dataloggers

There is a group of IMUs that provides information similar to that offered by current commercial dataloggers (i.e., raw inertial data) but, furthermore, the manufacturer offers a mechanism to program the system’s microcontroller so that the user can embed an algorithm for different applications on robotics, navigation or activity recognition. However, this kind of systems are available in the market as development kits implying the need of an external power supply, the enclosure for autonomous use is not available and the microcontrollers have limited resources. For instance, the 6-DOF IMU Shield from DFRobot is a small system on board with a triaxial accelerometer and gyroscope that can be connected to an Arduino© platform. The web page of the product provides a code that can be downloaded to the Arduino© platform’s microcontroller and enables the user to embed any algorithm. Similarly, FreeIMU, which is a 9-axis IMU [34], is connected to the same Arduino© platform as the previous one and has an Atmega368 microcontroller with a 32 kB of flash memory and 2 kB of random access memory (RAM) capacity.

In contrast, Sparkfun’s UDB5, which has the microcontroller integrated within the board, is a light and small system-on-board with a dsPIC33FJ256GP710, which has 256 kB of flash memory and 30 kB of RAM memory. This system allows connecting an external GPS providing even more information to the user. Another example of open source datalogger is the x-IMU from IO-Technologies [31]. This system-on-board platform is a 10-axis IMU (including a barometer) that incorporates a dsPIC33FJ256GP804, which is a microcontroller with 128 kB of flash memory and 16 KB of RAM. All these datalogger systems need a wire or an external complementary board in order to send data to a physical storage unit. Likewise, an external complementary board is needed if the user wants to send or receive commands wirelessly. Moreover, no battery system or enclosure is provided by manufacturers and, therefore, it is difficult to implement a portable inertial system for users.

### 2.3. Parkinson’s Disease Monitoring

Currently, there exist three main systems that are commercially available and provide information on PD symptoms: Kinetigraph™ from Global Kinetics [35], and Kinesia ONE and Kinesia-360™ from Great Lakes NeuroTechnologies [36].

Kinetigraph™ is a single wrist-mounted system based on a triaxial accelerometer with a sampling frequency of 50 Hz and it has a full-scale of ±4 G [35,37]. The system can record data for 10 days, and then the user must send this data to a company server and, subsequently, receives a detailed report. The employed algorithm is based on the assessment of frequency features from harmonics starting at 0.2 Hz to 4 Hz through features extracted from band’s power spectral density, for example, the average and the maximum peak within the aforementioned band [37].

Kinesia-360™ is a dual inertial system that consists of two sensors, one mounted on a wrist and another one placed in patients’ ankle. Both devices include a triaxial accelerometer and gyroscope. Kinesia system also includes a mobile phone application, which is used as an interface between the system and the user. Furthermore, this data is also sent to an external server in order to ensuring data protection. Kinesia system provides information about tremor [38], dyskinesia [39] and mobility based on temporal and frequency features. The outcomes of the algorithm are gathered in a file, which is updated every 2 min with a new result. Great Lakes NeuroTechnologies has also released Kinesia ONE, which also provides results in form of indexes from bradykinesia, dyskinesia and tremor. However, this system is thought to be used under scripted tasks following an app provided by the same company [40]. This system is composed of a small inertial system which is worn on the finger; this way, after executing specific tasks such as finger tapping, it enables the objective quantification of some PD symptoms such as bradykinesia.

The device presented in this paper is capable of detecting different symptoms of PD (bradykinesia, dyskinesia, ON/OFF fluctuations and freezing of gait) using only a single waist mounted system. As previously described, this device has been designed as a new version of a previous sensor and to fulfil a set of requirements set by a clinical study. In addition to providing PD symptoms results, the 9 × 3 enables capturing raw data for being analysed later. This constitutes a useful tool for researchers and clinicians, who can use raw data to build machine learning models and, furthermore, models can be implemented in real time enabling an objective monitoring of symptoms and actuate instantly when an event or the appearance of a specific symptom is detected.

## 3. Requirements of the System

This section presents the system’s main requirements given the context of use of the presented device. However, this system is also capable of covering other scenarios different to the described clinical protocol. Within the MASPARK project, the clinical protocol establishes that the device has to be used both in laboratory settings and at patients’ home. Thus, the system must accomplish different requirements imposed by the nature of the experiments. More specifically, in MASPARK project, patients will use the system during 30 days at home without any assistance. Additionally, the clinical protocol requires that the system should work during at least 10 waking hours without charging the battery.

### 3.1. Clinical Protocol

The MASPARK clinical study has been approved by the local ethics committee and has been divided into five stages. The clinical study’s main goal is to analyse the efficacy of administering automatically rhythmic auditory stimulation (RAS) to PD patients when FoG episodes and bradykinetic gait appears; furthermore, dyskinesia detection is also tested in order to perform a complete map of PD symptomatology. According to previous studies [3,41], bradykinesia is a symptom which is manifested differently in each patient. A similar situation occurs with FoG [42], which has been shown to be user dependent. Thus, the algorithms devoted to detecting these symptoms have to be personalized and, hence, the clinical protocol includes different stages to this end. Below, the five stages of MASPARK clinical shown in Table 2 are briefly described. Note that, as detailed in the experiments section, this paper analyses the 12 PD patients data related to Stage 1.

- Stage 0: In this stage, a baseline exploration is performed by clinicians. They will ask patients to come into doctor’s office to receive an information sheet and the consent form. Then a series of tests are performed and questionnaires are filled in with the aim of mapping the severity of the disease symptoms by means of the UPDRS [43] and Hoehn & Yahr scale [44]. A total of 25 patients will be recruited in two parts (12 patients the first part, 13 patients the second part) for the data collection after providing informed consent according to the Declaration of Helsinki.
- Stage 1: This stage’s main goal is devoted to acquiring inertial data during patients’ activities of daily living in order to adapt two personalized machine learning classifiers to detect bradykinetic gait and freezing of gait. Thus, in this stage patients receive the inertial system and wear it during 3 days at waking hours, at least 10 h, in which the system acquires inertial data at 50 Hz. The fourth day patients go to doctor’s office, where he/she executes, with the sensor in the waist, a series of tests also video-recorded in order to have a gold-standard. The aim of this experiment is to perform a laboratory test of the required real-time algorithms from the inertial system, so that bradykinetic gait and FoG real-time detections are compared to the video recordings. Experiments and results of this test are shown in Section 6 and Section 7, respectively.
- Stage 2: The main objective in this stage is to personalize the RAS system according to patients’ preference and test the system under laboratory conditions. One of the main advantages of the MASPARK system is the capability of providing an actuation once a FoG episode or a bradykinetic gait state is detected. This actuation relies on RAS which enhances gait whenever these symptoms appear [45,46]. Hence, the user is invited to test the headset in laboratory conditions. The headsets will produce different RAS and clinicians will evaluate which one is more effective.
- Stage 3: This stage is dedicated to test the effectivity of the RAS cues in the ADL of patients administered through the MASPARK system (Inertial sensor, smartphone, and headset). Patients will use the 9 × 3 device in two periods of 4 days each, a 4-day period with the actuating system enabled and the other period being the system disabled. Different measures, such as the number of FoG episodes, will be compared among both periods to determine the effectivity of the approach.
- Stage 4: In this stage, patients will use the system during 30 days continuously. At night, patients will charge the 9 × 3 with a standard mobile phone charger (micro-B USB connector). The system will send RAS to the headsets when it detects a FoG or bradykinesia episode, as in Stage 3. The main goal is to test the system in long periods, check usability and analyse patient’s quality of life before and after the use of the system over a longer period.

Given the clinical protocol, there are different requirements that the 9 × 3 system should undertake. The minimum duration of the system is set to 10 h (waking hours). Thus, and to be sure that data are not lost (in case patients forget to charge the sensor device), it is required to capture data continuously during, at least, 3 days (corresponding to the 3 days the patient should wear the system at Stage 1) without charging the battery. The same duration is required when the system only computes the embedded personalized algorithms (i.e., FoG and bradykinetic gait detection). Wireless communication is necessary for two reasons; first, when a FoG or bradykinetic gait episode occurs so that a message is sent to the mobile phone to activate RAS. Second, it is mandatory for the IMU to be able to get the current time from the smartphone so that doctors could know when symptoms occur. Additionally, a standard and simple charging system such as the employed in smartphones is required.

Furthermore, due to the described clinical protocol conditions, the 9 × 3 has to accomplish some prerequisites regarding the size and weight taking into account that the system should be worn close to the human centre of mass. In this sense, some studies have been found related to the position to wear a sensor. According to Yang et al. [10] and Gjoreski et al. [47], the waist location is interesting from the biomechanics point of view, since it is close to the centre of mass of the human body, and it also provides good ergonomics to patients. Furthermore, in the same line, Mathie et al. [48] conducted a survey that confirmed the lateral of the waist above the iliac crest as the most comfortable site to place an IMU. Therefore, considering this rationale, the waist has been chosen as the most suitable place to wear the 9 × 3, which was also chosen in the previous version of the device.

### 3.2. Hardware Requirements

Regarding the hardware requirements, the system must contain, at least, one accelerometer in order to register measurements related to movement. Furthermore, it is considered a benefit to work with different measurement ranges (i.e., full scale values) and sensitivities to accurately analyse those movements with very low acceleration (micro-movements), and also to study very rapid and abrupt movements such as falls without any saturated signal. Thus, it might be necessary to include two accelerometers configured at different sensitivities to cover these movements. Moreover, a third accelerometer is required to use special features such as waking up the system when a movement is detected. In this sense, this accelerometer will allow the system to sleep in those periods of time with absence of movement. Once a motion is detected, an interrupt will be activated by this accelerometer, which will wake up the microcontroller and execution of algorithms will be resumed. On the other hand, barometers are increasingly being used in the field of activity recognition due to its capacity to detect small changes in altitude [15,16,33]. Hence, authors consider interesting to also include a barometric sensor since the use of this sensor has not been explored in patients with PD.

Regarding the usability specifications, the device is required to feature a single switch, a buzzer, and a LED. The button will allow the user to switch on and off the system and the LED will show the working mode and the state of the battery level. The buzzer is required in order to warn the user in case of critical battery level or other error concerning communications, sensors or algorithms.

In order to perform an estimation of the capacity of internal memory needed to gather signals during a week, it has to be taken into account the amount of information gathered every second. The device is required to gather the following information:

- Accelerometer 1 (axes X, Y and Z): 12 bytes.
- Accelerometer 2 (axes X, Y and Z): 12 bytes.
- Accelerometer 3 (axes X, Y and Z): 12 bytes.
- Gyroscope (axes X, Y and Z): 12 bytes.
- Magnetometer (axes X, Y and Z): 12 bytes.
- Barometer: 4 bytes
- Device temperature (accelerometers 1, 2, 3, and barometer): 16 bytes
- Battery status: 4 bytes
- Real-time registers (year, month, day, hour, minute, and second): 6 bytes
- Sample counters: 4 bytes

Hence, a total of 94 bytes per frame is, at least, required to be logged at every acquisition. In order to adapt the frame and having into account that some results can be included within the logged frame, it is required to work with a 128-byte frame. According to Zhou et al., sampling data at 40 Hz is enough to capture the main human movements [49]. In this work, it is considered as a requirement that the system captures inertial signal at least at 50 Hz. Hence, considering a 128-byte frame at 50 Hz, the system needs a memory of 552.96 Mbytes every day. The system needs, therefore, a microSD card to gather the information corresponding to the 3 days (1658.9 Mbytes).

On the other hand, as justified in Section 3.1, a Bluetooth module is also necessary for wireless communication purposes. Additionally, the external connection should be performed similarly to a standard mobile phone, that is, with a micro USB type B.

Finally, a microcontroller is necessary to manage all peripherals. The microcontroller requires to support all the external interrupts (sensors’ *data ready* output, switch button, charge status), at least two I^2^C buses (power system and inertial sensors), Secure Digital Input Output (SDIO) communication bus, General Purpose Input-Out (GPIO) ports, analog-to-digital converter (to read the battery level) and a pulse-width modulation module to provide a modifiable pulse signal to the buzzer. Furthermore, the microcontroller has to include a low-power mode in order to drastically reducing the consumption when no action on calculus is executed. An external interrupt (accelerometer *data ready*) will be the only event that will be able to wake up the system. Finally, a real-time clock system is included with the aim of keeping the current date and time.

Regarding the amount of memory employed within the microcontroller, there are some parts of the embedded algorithms that need the use of the RAM memory. The memory size needed is difficult to be calculated since it relies on its local usage in specific parts of the program. An estimation of the memory usage in a high-performance part of the program is 60 kB: thus, the microcontroller has to include a RAM memory of 128 kB [50]. Another part of the algorithms (classifier models) can be stored within the flash memory. For example, the FoG classifier, which is based on Support Vector Machines (SVM), is the biggest classifier model concerning memory capacity. It has 211 support vectors and 27 features. This number implies a memory consumption of 211 support vectors × 27 features × 4 bytes each float = 23 kB. Given that the algorithmic module (Section 5) contains two SVM modules and the code has to be allocated there as well, the flash memory capacity of the microcontroller has to be of, at least, 512 kB.

## 4. 9 × 3 Hardware Architecture

Provided the requirements from Section 3, this section details the 9 × 3-IMU’s hardware. First, the microcontroller and its main surrounding peripherals are described, then the inertial system is explained and finally, it is briefly described the functioning of the communication module, which includes the Bluetooth module and the microSD card.

### 4.1. Microcontroller

**Figure 1:**
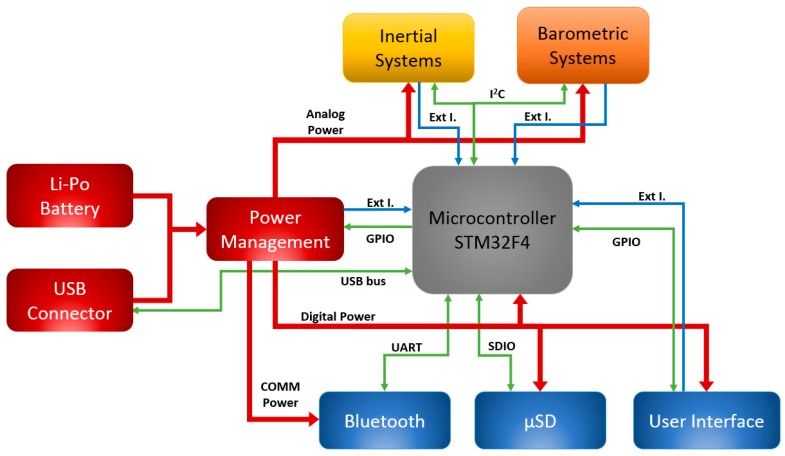
9 × 3 general structure with main connections.

The microcontroller is the core of the system and its main goal is to manage all the peripherals and devices that constitute the 9 × 3 in addition to executing the algorithms embedded. In Figure 1, the main scheme of the system is shown, reporting the main communication lines between the components.

The microcontroller is an STM32F415RG from STMicroelectronics, a Cortex™-M4 CPU with floating point unit [51]. The microcontroller works with a clock frequency that can reach 168 MHz and provides a computational speed of 210DMIPS. There is an internal flash memory of 1MB and 192 kB of RAM.

The Direct Memory Access (DMA) module enables the microcontroller to perform calculations while the peripherals are exchanging data autonomously. The microcontroller has up to three I^2^C buses, being two of them used to lessen the data transfer burden in the I^2^C bus in case different accelerometers and barometers send raw data at its maximum speed. Universal Asynchronous Receiver Transmitter (UART) is employed to control the Bluetooth module so that messages from the smartphone are sent and received. Communication with the microSD card is performed through the SDIO peripheral. Although the USB peripheral is used to charge the battery, it is also used to read the content of the microSD card through the USB On-The-Go Full Speed bus. On the other hand, the microcontroller contains 17 timers. Some are employed to control timeouts and the pulse width generator employed for the buzzer activation. Furthermore, the microcontroller is capable of managing up to 16 external interrupts, which might be used to wake up the system (e.g., accelerometer *data ready* to be read).

Overall, the microcontroller outperforms the one used in the former IMU as shown in Table 3. The new microcontroller has less power consumption, larger memory and more peripherals. In addition, the microcontroller includes a floating-point unit that enables easily computing algorithmic models.

### 4.2. Sensors

**Figure 2:**
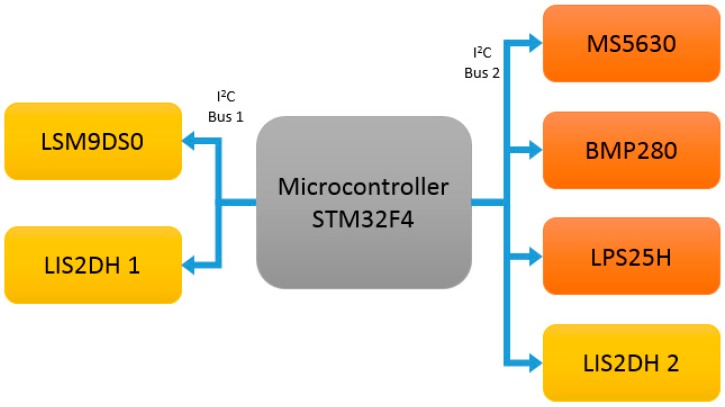
Main connection between the inertial sensors and the microcontroller.

The 9 × 3 contains several inertial and barometric sensors. The inertial sensors are the LSM9DS0 [52], which is a 9-axis system composed of a triaxial accelerometer and magnetometer module, and a triaxial gyroscope module. Furthermore, two LIS2DH [53] have been included in order to satisfy the stated requirements in Section 3. The LIS2DH is a triaxial accelerometer designed to be embedded in small devices since its package dimension is 2 × 2 × 1 mm^3^. The included barometric system is composed of three high accuracy barometers: BMP280 from Bosch [54], LPS25H from STMicroelectronics [55], and MS5637 from TE Connectivity [56]. According to Section 4.2.3, it must be noted that it is not required to embed three barometers; however, the main purpose of embedding three barometers into the 9 × 3 is for analysing and comparing the performance of different commercial barometric sensors. However, as a final device for detecting PD symptoms or activity, it is required that at least one barometer is embedded. The sensors are distributed and connected to two I^2^C buses as shown Figure 2, releasing, thus, high burden data transfer in one bus.

#### 4.2.1. LSM9DS0 Description

The LSM9DS0 is a *system-in-package* that contains two modules, one contains an accelerometer and a magnetometer system, and the other contains a gyroscope forming, all together, a 9-axis system in a LGA-24 4 × 4 × 1 mm^3^ device [52]. The sensors have a 16-bit output provided through an I^2^C or SPI communication. This system has four interrupt lines (two for each module) with different configurable functionalities such as *data ready*, *buffer ready*, *acceleration threshold* and *specific orientation*, among others. Furthermore, the LSM9DS0 incorporates an internal memory for buffering, which allows keeping all the system in very low power consumption mode and only running once the buffer is full; then, the system transfers the buffered data to the microcontroller.

The accelerometer allows diverse configurations for different purposes. For example, the output data rate is programmable from 3.125 to 1600 Hz having the possibility to include an internal low-pass filter to remove noise from 50 to 773 Hz. The accelerometer can also be configured regarding the range of measurement (±2 G/±4 G/±6 G/±8 G/±16 G, where 1 G = 9.81 ms^−2^) modifying, hence, the sensor sensitivity. The magnetometer, which is included in the same internal module as the accelerometer, provides also different configuration possibilities such as full-scale selection (±2/±4/±8/±12 Gauss) and output data rate from 3 Hz to 100 Hz. The gyroscope is included in the same package and its resolution and data rate can be programmed at 245 degrees per second (dps), 500 dps or 2000 dps and the data rate can be programmed from 95 to 760 Hz, respectively.

#### 4.2.2. LIS2DH Description

The LIS2DH system is a 12-bit triaxial accelerometer manufactured by STMicroelectronics [57]. This system is a low cost/power accelerometer embedded in an LGA-14 and 2 × 2 × 1 mm^3^ package. It provides an output data rate from 1 Hz up to 5.3 kHz. The accelerometer has two interrupt lines for different purposes. For instance, the accelerometer system is able to generate an interrupt when data is ready to be sent to the microcontroller. On the other hand, there is a set of different configurations. One of the configurations might be for waking up; once the sensor detects movement the accelerometer sends an interrupt to the microcontroller. Otherwise, the accelerometer keeps the system in sleep mode with a very small power consumption. Another possible interrupt can be the detection of a specific orientation of the system (with respect to gravity) so that an interrupt is generated when the accelerometer detects it. In addition, an embedded function to detect double click o single click may be enabled. Finally, a free-fall event has also been included. The system can be configured with a range of ±2 G/±4 G/±6 G/±8 G/±16 G and data can be provided at each sample or after filling an internal FIFO buffer.

#### 4.2.3. Barometric System Description

Three barometric pressure sensors have been integrated within the 9 × 3 system in order to analyse their performance among them. It must be noted that embedding three barometers is considered merely exploratory, not being required for the MASPARK project. MASPARK project only requires one barometer sensor in order to investigate its benefits in the monitoring of PD symptoms through the analysis of activity recognition, falls and posture transitions. Thus, the remaining two barometers are disabled and not used for the purposes of the project.

Within the 9 × 3, some of the most frequently used and with higher performance barometers have been included: LPS25H from STMicroelectronics, MS5637 from TE Connectivity and BMP280, from Bosch, which are compared in Table 4.

The three barometers exhibit interesting features. They provide a very reasonable barometric range, showing the ability to be employed at any place on Earth (Everest Mt. 8848 m = 337 mbar, sea level = 1013 mbar) [58]. Regarding the quality of the signal, LPS25H is the most accurate concerning the resolution of the sensor; however, it presents almost ten times the BMP280 noise. As a result, BMP280 is the most accurate one. Among the three barometers, the BMP280 is the smallest sensor and the one that consumes less energy. The three sensors provide a compensation system with temperature, internal filters and present sufficient data rate, being the MS5637 the quickest with 60 Hz.

Within the framework of the MASPARK project, it has been considered to employ the BMP280 due to its good resolution, sufficient range, and notable accuracy. The data rate is enough in order to capture a fall or a posture transition, which are movements that have an accelerometry frequency response below 0.68 Hz [59]. The most important feature, thus, is the noise, which is the lowest of the compared sensors and allows then to observe a signal with good quality in movements where the altitude is involved (e.g., fallings, stand to sit or sit to stand posture transitions).

### 4.3. Communication and Storage Units

The 9 × 3 IMU includes two modules for sending and storing information. The first one is a Bluetooth module (WT12A manufactured by Bluegiga), which has two main objectives: first and mainly, provide the signal to the smartphone which will activate the headset for the RAS. This stimulation will be activated when the 9 × 3 detects a FoG episode or a bradykinetic gait. Furthermore, the second objective is to synchronize time and date with an external device such as a computer or a smartphone. This will allow locating the time when a symptom has been detected and the time it has lasted. The second module is a microSD card, which has been included to store all the available information autonomously. It allows the researchers accessing to all the stored raw data and, in addition, it permits the clinicians to download the entire symptoms history and activity of the patient during the capture. Hence, the microSD card is the best option since it is very small and cheap, and it can store a large amount of information. The internal firmware of the 9 × 3 device has been prepared to work with FAT32 system being compatible with cards with more than 2 GB.

### 4.4. User Interface

**Figure 3:**
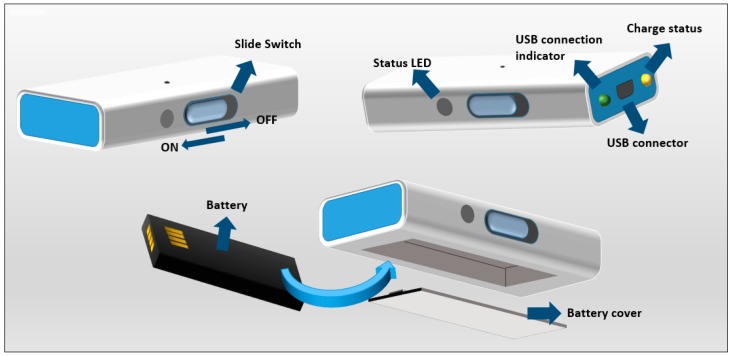
9 × 3 parts.

The user interface of the 9 × 3 has been designed with the aim of maximising the ease of use. Thus, the user can only switch off and on the sensor with the slide switch as shown in Figure 3. The sensor includes two LEDs that indicate the wired connection status. Meanwhile the *USB connection indicator* turns on when a USB connector is plugged, the charge-status LED indicates if there is any charge in process. When the charging process is completed, this LED turns off.

**Figure 4:**
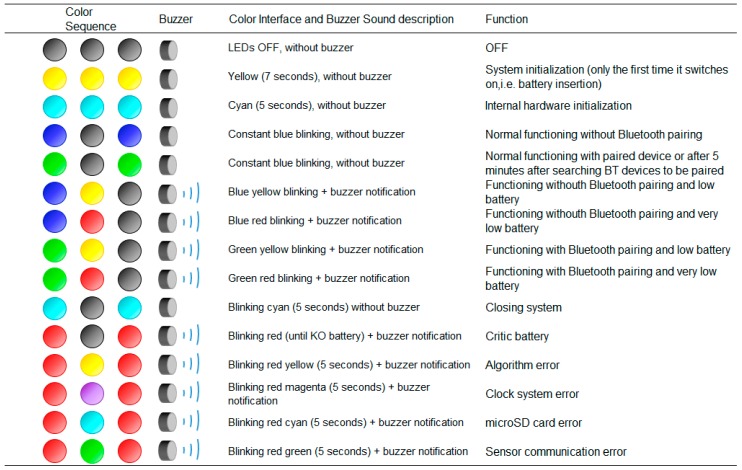
Status LED colour codification.

The *status LED* provides information of the different processes of the device as shown in Figure 4. There are different working modes, but there is also an error coding in case the system presents a failure related to clocks, microSD writing process, communications or algorithms. The buzzer helps the user to identify the status of the system, for example, when battery charge is low.

## 5. Firmware

This section first reviews the management of the microcontroller peripherals. The firmware system is composed of different concurrent processes that are executed conditioned by specific events. Second, the implemented algorithms for detecting PD symptomatology are briefly described, emphasizing their computational relevance.

### 5.1. Peripheral Microcontroller Management

In this section, the peripheral management policy is described from the point of view of the microcontroller. There are two main functional blocks, the hardware control block, which is devoted to managing all the surrounding peripherals, and the algorithmic block, which is dedicated to controlling the steps of the embedded algorithms. With the aim of describing them appropriately, the firmware has been subdivided into 8 blocks, belonging 6 of them to the hardware control block (data acquisition, microSD data control, Bluetooth communications control, user interface block, power control and system control) and the remaining one to algorithmic block (sample and window computation).

**Figure 5:**
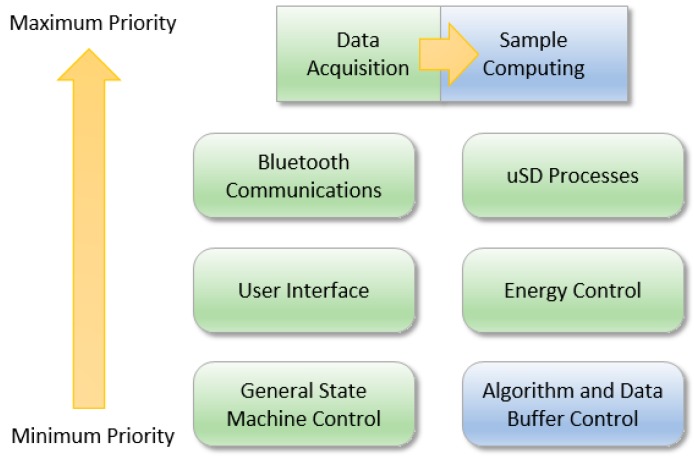
Firmware priorities composition.

Figure 5 shows the main blocks that represent the different processes that interact within the firmware. Green blocks are those corresponding to the hardware control and the blue ones correspond to the algorithmic block. They are sorted according to their priority of execution, being those on the top the ones with higher priority and those on the bottom the ones with lower priority. This priority enables to decide the process to be executed whenever there is a conflict. According to Figure 5, those blocks that are at the same level have the same priority, and only a higher priority task can interrupt them.

As Figure 5 shows, Bluetooth communications and microSD control processes have the maximum priority after data acquisition and the sample computing processes. These processes are asynchronous and relatively long with several internal states that depend on time constraints. In case these processes are interrupted, this could cause errors or information loss. Thus, a high priority is assigned to them. However, the data capture process (data acquisition and sample computing processes) can interrupt the microSD reading and writing processes due to two reasons. On the one hand, if the data-sample capturing process is interrupted, it would produce a loss of information. On the other hand, since the sample computing is fast, the highest priority is assigned to it and it has been considered to include a small computation at every new received sample.

The energy control process manages and determines when the system has to enter into low power modes. For example, when an inactivity period is given due to a wait response of some peripheral or when the system is waiting for the next sample, the microcontroller enters in low power mode. This block also controls battery state, indicating the battery level to users and stopping the system when a critical battery condition is detected. Furthermore, it enables and disables the internal linear regulators that control different parts of the circuit in order to save energy (e.g., Bluetooth regulator disabled when no command is expected from an external agent). The user interface system controls the two main feedback sources, which are, as previously shown, the RGB LED and the buzzer.

The algorithmic block and the system control are the processes with less priority; however, they control the execution of all processes of the system. Due to this, these processes are executed in the main thread of the system and they are designed to tidily handle requests produced by the remaining processes. An error control system has been also implemented to supervise buffer overflow, waiting times or excessive execution time at some critic processes, such as the sample computation. Note that when data capture mode is programmed, the algorithmic block is not executed. In this case, buffer management is different since the system stores within the internal memory the data from 3 accelerometers, a gyroscope, a magnetometer, a barometer, temperature, battery level and real time clock registers.

Once the 9 × 3 is switched on for the first time (when the battery is inserted), the system checks whether the USB is connected. If the device is connected to a computer through a USB, the system enters into a specific mode that allows the user to download all captured sessions. At the same time, the power system will enable the charge of the battery through USB. If the 9 × 3 is not connected to any computer, only the charging process will be executed. In contrast, if the system is not connected by USB, the General State Machine Control enables the device to capture inertial data or to compute algorithms.

### 5.2. Embedded Algorithms

In this section, the algorithms that can be implemented into the microcontroller are described. However, in the following sections of this work, only two of them, FoG and bradykinetic gait algorithms, will be presented as results since they are the main algorithms involved in the clinical study previously presented.

**Figure 6:**
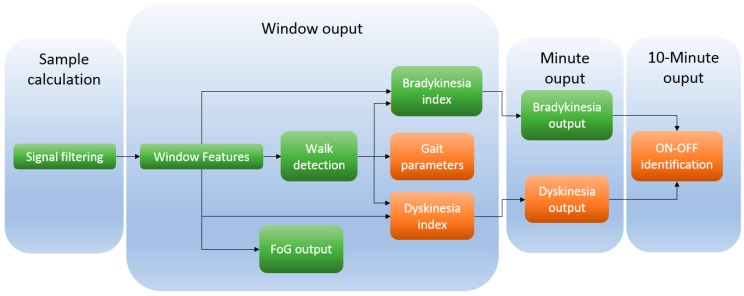
Structure of the algorithms that can be implemented within the 9 × 3. In green colour, those blocks that have been implemented and tested in this paper.

In total, the microcontroller has been programmed to implement five embedded algorithms: walking recognition, bradykinetic gait detection, dyskinesia detection, FoG episode identification and gait parameters extraction. These algorithms have been previously presented by the authors in other works [3,41,60,61,62,63]. With these classifiers, along with the gait analysis algorithm, an identification of the PD motor state can be performed. All these algorithms are composed of other computational processes that are listed in Table 5 and organized as established in Figure 6.

According to Table 5, the 9 × 3 provides two different outputs that might activate RAS: bradykinetic gait episode (minute output) and FoG episode (window output). There is an output that is given every 10 min, which is the ON-OFF state. This algorithm relies on the bradykinetic gait and dyskinesia output. Since these symptoms are motor symptoms that might be manifested during several hours, it is not necessary to provide a continuous output. Thus, a 10-min output is enough in order to monitor this symptom [3,41].

However, previously to this 10-min output, there are four different levels of implementation and computation of the samples acquired at 40 Hz. According to Figure 6, the calculations of the 5 algorithms are divided into four temporal levels (gait parameters extraction, freezing of gait, bradykinesia, dyskinesia, and ON-OFF state). The first level consists of acquiring the signal and applying the corresponding filter. In our previous works [3,41,61,65], sampling frequency was 40 Hz. This frequency was selected due to two reasons: the first one, it has been shown that 40 Hz is enough to measure human movement [49]; second, due to the maximum frequency of interest within the PD movements, which is 20 Hz for walking activities [61]. Regarding the filter, a 2nd order Butterworth low-pass filter has been employed to remove the noise of the signal.

After collecting a signal window, feature extraction is performed with the aim of characterising some typical signal behaviours. The extracted features are, firstly, temporal characteristics such as mean, standard deviation, range, signal magnitude area, signal correlations, distribution analysis like skewness, kurtosis; and secondly frequency features are used, such as energy and spectral density in specific bands. From these features the bradykinetic gait [41,64] and dyskinesia indexes are extracted [61], as well as if the person is walking based on an SVM model [41,64,66], and the FoG detection output [60]. The calculated index for dyskinesia and bradykinesia, however, might be dismissed given that the person might not suffer neither dyskinesia nor bradykinesia in a certain moment of the day. It is necessary then, to ensure that at least a minimum number of windows containing the symptom exist in order to evaluate it. Hence, the evaluation of consecutive output of bradykinesia, and dyskinesia determine the final symptom output every minute. Finally, and given that the ON-OFF state is a motor state that lasts several minutes, it has been considered to provide an output every 10 min [66].

The FoG algorithm provides an output every window. However, FoG is not necessarily dependent of the ON or OFF state [67,68]. This has been reported to be user dependent and current algorithms show a significant improvement when the algorithm is personalized [42]. Thus, depending on the user, FoG might be an indicator of the ON or OFF state but, in this work, FoG outcome does not influence this output.

**Figure 7:**
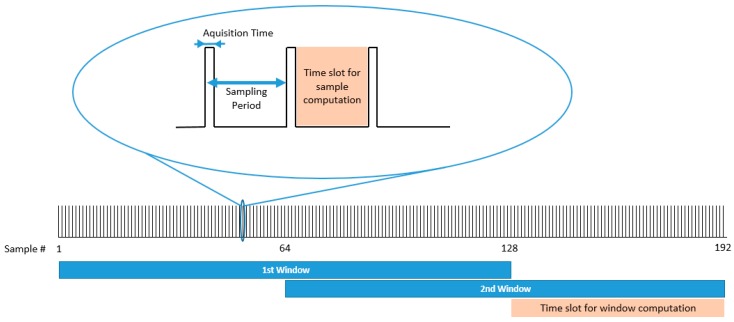
Temporal representation for acquiring and computing the extracted features.

Figure 7 shows the temporal organisation between the data acquisition and the feature extraction. The time interval between sample acquisitions is the available time for computing the algorithms associated with calculations. It must be noted that the computing time for each sample cannot be longer than the available time among samples acquisition, otherwise, calculation actions would be missed and, thus, errors would appear.

In addition to the temporal organisation of the algorithms, although it is not interesting to describe each one of the implemented classifiers (Table 5) since they are reported in other papers [3,41,60,61,62,63], it is interesting to describe some specific calculations that are implemented into the microcontroller. Below, the most relevant parts are described, which are the filtering, some temporal and frequency feature extraction and the SVM. In the design of the 9 × 3 device, consumption constraints have been the most important ones in order to meet autonomy requirements imposed by the final scenario. Thus, during the algorithmic design process, the memory resources and the number of computational operations required by the algorithms were minimised.

According to Figure 6, signal filtering is the first computation performed within the structure. The filter proposed is a second order low pass Butterworth filter. The filter allows to remove the noise not related to human movement. The Butterworth filter is represented by two vectors *a* $\in R^{2}$ and *b* $\in R^{3}$ that filter the signals with a low computational cost as the new filtered value consists of the combination of the current sample and the last two filtered and non-filtered samples, i.e., for X axis $x_{i}^{\prime }=\sum_{j=1}^{3}b_{i}x_{i-j+1}-\sum_{j=1}^{2}a_{i}x_{i-j}^{\prime }$ where $a_{i}$ and $b_{i}$ are the Butterworth coefficients obtained and $x_{i}$ is the current sample.

Once a data window is completed, window features are extracted. There are some features that are employed in all the algorithms shown in Table 5. Among them, mean and standard deviation, that are defined by ${\bar{x}}^{W}=\frac{1}{N}\sum_{i=1}^{N}x_{i}^{w}$ and $\sigma x^{W}$ = $\sqrt{\frac{1}{N}\sum_{i=1}^{N}{\left(x_{i}^{W}-{\bar{x}}^{W}\right)}^{2}}$, respectively, where N is the number of samples in a window, and W is the current window number.

The Short-Time Fourier Transform (STFT) is one of the main features employed within the algorithms. It is composed of a set of complex values $X_{1}^{W},\ldots ,X_{N}^{W}$ which are obtained following the next equation:

(1) $$X_{h}^{W}=\overset{N}{\underset{n=1}{\sum }}x_{n}e^{-i2\pi h\frac{n}{N}}$$

where h=1,…,N.

The STFT’s output is a set of N complex numbers, which represent the amplitude and the phase of the harmonic. In this work, only amplitude is interesting. However, not all the harmonics are interesting, for example for posture transitions only those harmonics from (0, 0.68] Hz are employed. Then, the energy spectral band of the harmonics of interest, or a ratio between two different bands are used as a feature vector for the classifiers.

For FoG detection, other features are calculated such as Pearson’s correlation coefficients, e.g., for the X and Y axis: $\rho_{x,y}^{W}=\frac{\sum_{i=1}^{N}\left[\left(x_{i}^{W}-{\bar{x}}^{W}\right)\cdot \left(y_{i}^{W}-{\bar{y}}^{W}\right)\right]}{N\cdot \sigma x^{W}\cdot \sigma y^{W}}$, and skewness e.g., for the X axis: $\gamma_{X}^{W}=Ε\left[{\left(\frac{x_{i}^{W}-{\bar{x}}^{W}}{\sigma x^{W}}\right)}^{3}\right]$. Regarding the assessment of bradykinesia, the symptom evaluation is widely described in [64,66]. The feature extraction consists of extracting energy spectral density of a concrete band for detecting gait. Then, after asserting that there is a continuous gait cycle, bradykinesia severity is determined. On the other hand, gait parameters are obtained following the method proposed by Sayeed et al. [62]. Finally, dyskinesia detection is mainly based on frequency features and the evaluation of different frequency bands in order to estimate its severity [61]. With the results of these algorithms, an evaluation of PD’s fluctuations is realized according to Pérez et al. method [66] with the aim of determining the ON and the OFF state.

After extracting all the features, specific classifiers are executed. As shown in Table 5, SVMs are employed to determine whether a walking activity or a FoG episode has occurred in the current window. The implementation of the fast-forward phase of an SVM is given by the following equation:

(2) $$f\left(x\right)=sign\left(\overset{l}{\underset{i=1}{\sum }}\alpha_{i}y_{i}\cdot e^{\left(-\frac{{\left\|x_{i}-x\right\|}^{2}}{2\sigma^{2}}\right)}+b\right)$$

where the expression $\|x_{i}-x{\|}^{2}$ denotes the squared Euclidean distance from two vectors being $x_{i}$ the i-th support vector and x the pattern to be evaluated. $\alpha_{i}$, $y_{i}$, σ and b parameters are constants extracted from the trained SVM model.

The rest of algorithmic blocks were implemented by means of decision trees or threshold based classifiers. The fluidity computation score is based on frequency features and is compared to a threshold trained by means of a leave one out process [41,64].

## 6. Experiments

This section details the experiments performed with the 9 × 3 in order to test this device in Parkinson’s disease patients with two main design goals: real-time detection of symptoms and data capture. First, we describe the experiments performed within the laboratory in order to test the device under controlled environments and to measure parameters such as autonomy, consumption and execution timings with the embedded algorithms. Then, the experiment undertaken with PD patients is described, in which the outputs of bradykinetic gait and FoG algorithms (both embedded in real time) are evaluated.

### 6.1. Hardware Experiments

The hardware tests conducted aim to gather experimental data regarding the consumption, autonomy, and performance of the device in terms of execution time of the algorithms. In total, 4 tests are done to evaluate different performance parameters of the device:

- Test 1: Consumption evaluation
- Test 2: Autonomy evaluation (Algorithms)
- Test 3: Autonomy evaluation (Data capture)
- Test 4: Timing evaluation

The main goal of the Test 1 is to empirically determine the duration of batteries by analysing its instantaneous power consumption. In order to measure consumption, an Agilent Technologies© (now Keysight Technologies©, Santa Rosa, CA, USA) multimeter 34405A model has been used [69], and the power was supplied by a VARTA© Lithium Polymer (LiPo) battery with a capacity of 1200 mA/h (EZPack L) [70]. In order to perform the consumption data capture, a computer with Windows 7 OS and the software BenchVue Platform from Keysight Technologies© is employed through a USB wire connected to the multimeter, enabling a continuous consumption acquisition.

In this first test, we measure the average power consumption during one hour with the following five conditions, which are the hardest ones regarding the device autonomy since all the peripherals are being used:

- Sending a message through Bluetooth every 15 min to ensure Bluetooth communication between the smartphone and the 9 × 3 device.
- Storing algorithmic information within the microSD every minute.
- Storing date, time and battery level every minute.
- Computing algorithms at each window of data (FoG and bradykinetic gait). It requires windowing, filtering, feature extraction and SVM classification in real-time.
- Sampling data frequency set to 40 Hz.

Test 2 has the same configuration than Test 1; however, the main goal is to determine the device’s real autonomy (measured in hours) while executing real-time algorithms according to the MASPARK setting. The third test is devoted to analyse the autonomy of the system with the Data Capture program in the 9 × 3. We have established the following conditions for this test:

- Receive date and time at the beginning from another device via Bluetooth.
- Store inertial data and other parameters (3 accelerometers, gyroscope, magnetometer, barometer, temperature, sample counter, battery level, date and time) within the microSD at 50 Hz.

Finally, Test 4 was performed in order to measure the amount of time needed to execute the algorithms. This test has the same configuration than Test 1 by executing the algorithms for detecting FoG and bradykinetic gait, and, at the same time, writing the results in the microSD card. To appropriately measure time of execution, a general purpose input/output pin was employed: before the task began, the pin was set to ‘1’; similarly, when the task ended, the pin was set to ‘0’. To measure the time required by the microcontroller to execute the algorithms, an oscilloscope Lecroy Wavesurfer™ 44MX was employed. 

### 6.2. Experiments in the Clinical Environment

In this experiment, the system has been tested by 12 PD patients as part of the MASPARK clinical protocol (see Stage 1 from Section 3.1). The aim of this test is to evaluate the real-time detection algorithms in the laboratory, through a protocol designed to elicit FoG episodes and bradykinetic gait.

In order to elicit FoG episodes, the experiment is based on Schaafsma et al. tests [67], where patients walk 20 m after standing up from a chair and also perform turns in both directions. Patients also pass through a narrow space and end the test sitting down. All these circumstances, according to Schaafsma et al., are situations where patients are susceptible to manifest FoG episodes. On the other hand, for eliciting bradykinetic gait, Schaasfma test is repeated twice; firstly, before the patient takes her/his first daily medication dose and, secondly, once the patient has taken medication and its effect is evident. Tests were video recorded to obtain the corresponding gold-standard. Firstly, FoG episodes were labelled by clinicians, and overall gait was labelled either as bradykinetic or non-bradykinetic. These labels were compared against the output of the algorithms to determine their accuracy.

A total of 12 patients (four women, eight men) have participated in this experiment (first part of the clinical protocol described in Section 3). The mean age of the participants is 65.5 ± 6.259 years old, the mean Hoehn & Yahr stage is 2.95 ± 0.14 and the UPDRS score part III is 14.5 ± 10.88 in ON state and 26.75 ± 10.98 in OFF state (Table 6). The test was approved by the local ethics committee, all patients were diagnosed with PD according to London Brain Bank [71] and gave their written informed consent.

The FoG algorithm implemented within the microcontroller is presented in [63] and is user independent, being functional to any patient. The algorithm is based on an SVM model that has been trained and tested based on data from 21 PD patients at their homes. Patients performed a series of activities in which some were scripted and supervised by clinicians such as Timed Up and Go test, dual task, walk through a narrow space and turns. Other activities were scripted but the execution was entirely free, such as walking outside of the home and showing their home to clinics.

The algorithm to detect FoG consisted in the analysis of a triaxial accelerometer signal provided by the 9 × 3 on the waist. The signal was filtered and windowed, from which 55 features were extracted and set as the input of the SVM classifier to be trained. The model was trained through a leave-one-patient-out with an SVM based on a radial basis function kernel. This model was shown to enhance other FoG models but the computational cost was high. Therefore, the SVM model that is embedded within the microcontroller has been reduced in order to simplify the computational burden by means of Separable Case Approximation method [72].

The bradykinetic gait algorithm analyses patients’ gait according to the frequency content of strides. To this end, a SVM first detects gait, as previously presented. Gait cycles are then identified according to biomechanical properties of gait reflected onto the acceleration values, and then characterised through their frequency power density. The strides from each test (either with or without the medication) are characterised and averaged, so that two values are obtained by each patient representing each one of the tests. From them, bradykinetic gait is evaluated: if the average value is higher than a patient-dependant threshold, bradykinesia is assumed to be present; otherwise bradykinesia is determined as absent in that test. The patient-dependant threshold is obtained through the unlabelled data collected over three days (first part of Stage 1) by means of an ε-SVR model [64].

## 7. Results

**Figure 8:**
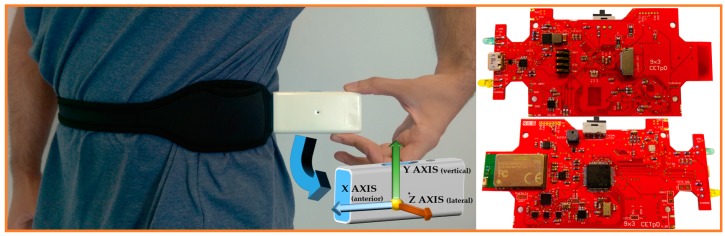
9 × 3’s location, accelerometer axes and top and bottom hardware view.

The 9 × 3 is a waist-worn IMU device capable of collecting inertial data and also suitable for the execution of embedded machine-learning based algorithms. Figure 8 shows the location and orientation of the 9 × 3 with the neoprene belt around the waist. The system fits the body, being comfortable for patients as shown in results of the REMPARK project (“Deliverable 9.2” available in “Public Documents” in www.rempark.eu), where only a 7% of patients considers the system is big for real life. Only a 4% thought the system weighs too much for daily use. As shown in Table 7, the system (with the enclosure) has a size of 99 × 53 × 19 mm^3^ and the whole system weighs 57 g (83 g with battery).

In the following subsections, results regarding its usage by PD patients, the power consumption of the system and the time the microcontroller needs to process the real-time algorithms are given.

### 7.1. Power Consumption and Autonomy Tests

**Figure 9:**
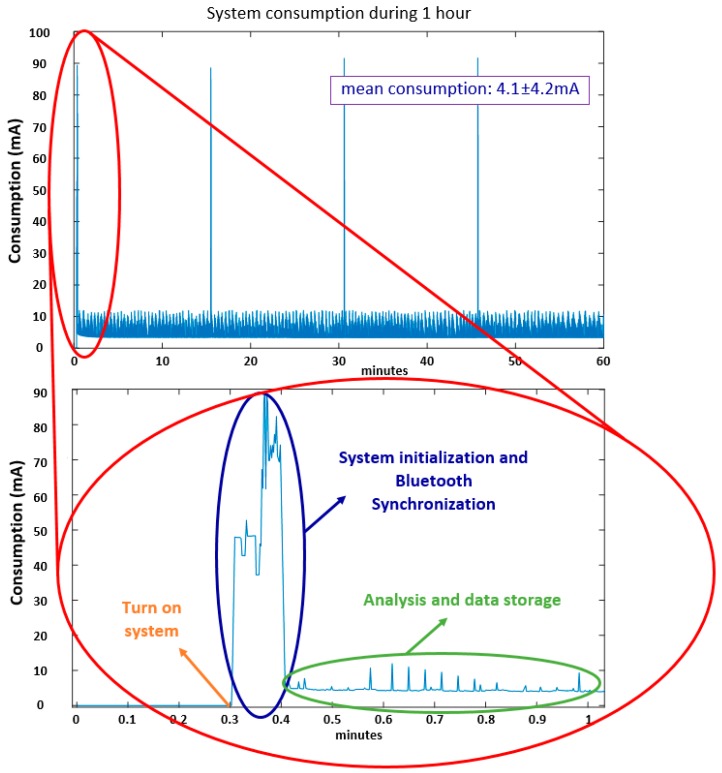
1-h consumption test.

In this section, we present the results obtained in Tests 1, 2 and 3 described in Section 6.1, where the power consumption of the device is computed in order to estimate the its autonomy. Figure 9 presents the consumption of the system during the one-hour test (Test 1). 

A peak in the power consumption can be appreciated at the initialization of the system, which is due to the Bluetooth communication when the system is synchronized with an external device. Depending on the conditions of connectivity with the external device, establishing a Bluetooth connection can take from few seconds to more than 5 min (our firmware limits this time to 5 min). In normal conditions, it only takes 5 s. The average consumption when there is no Bluetooth communication is 3.8 ± 1.2 mA. The average consumption of the system during the test has been of 4.1 ± 4.2 mA. The standard deviation is high due to some current peaks obtained when the microcontroller is sending the message to the mobile phone via Bluetooth. It must be noted that, according to MASPARK system requirements, the 9 × 3 should send a message to the mobile phone in order to send RAS to patients once a FoG or a bradykinetic gait is detected. This circumstance involves activating the Bluetooth and sending the message to the mobile phone, increasing the consumption.

The 9 × 3 employs a 1200 mAh LiPo battery. Under these conditions, the autonomy estimation is 29.6 days if the system works at waking hours (10 h a day). However, if the system works continuously, the estimation of system’s autonomy is of 12.3 days.

Regarding Test 2, the mean autonomy achieved with a 1200 mAh LiPo battery in five different devices was 230.9 ± 4.8 h, which corresponds to 9.6 days working continuously. Under these conditions, it is expected to work 23.09 days in waking hours. In Test 3, in which the device captures inertial signals at 50 Hz with the same battery, the data stored information of the three axes from three accelerometers, a gyroscope and magnetometer. In addition, data include 24-bit barometer values, temperature, battery level, RTC time and date, and a counter of samples. The autonomy of this test in five systems has been of 91.36 ± 6.65 h, which means almost four days of continuous functioning and more than 9 days of signals capturing during waking hours.

### 7.2. Timing Analysis of the Algorithms

**Figure 10:**
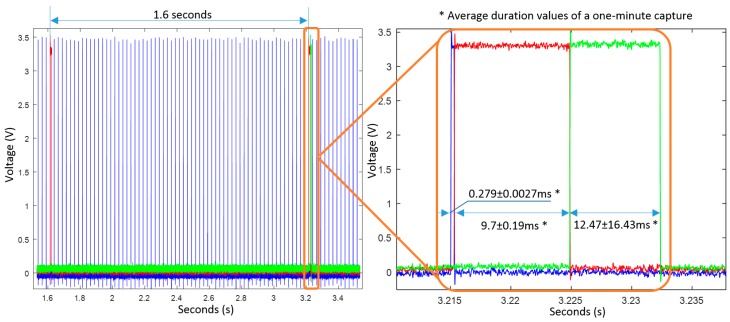
Process execution time. In blue, data acquisition and sample calculation time are shown. In red, window computation is represented, and, in green, stands for the microSD card writing process.

In this Section, we present the duration of the processes when the algorithms are executed (Test 4 Section 6.1). Figure 10 shows one of the obtained outputs. The microcontroller worked at 168 MHz and the sampling frequency of inertial data was 40 Hz, which is managed by the real-time clock peripheral (RTC). The RTC interrupts the program at every sample time. As shown in Figure 10, window computation is executed every 1.6 s. Once the RTC interrupts the program, a sample is received, filtered, and stored within the internal microcontroller memory. In case of completing a window, the program proceeds to compute the algorithms of bradykinetic gait and FoG to the signal contained in it. Figure 10 shows in red the duration of the computation process associated to the end of each window, which is about 20 ms. 

Each window lasts 3.2 s long and is 50% overlapped with the prior window, therefore, every 1.6 s a new computation is performed. Finally, in green colour, it is shown the time that takes the microcontroller to write all the algorithmic results to the microSD card. This time (12.47 ± 16.43 ms) is the time needed to write a 4 kB buffer of data. However, it must be mentioned that depending on the writing process of the microSD card (cluster and/or sector change), time spent to write the data buffer can reach up to 500 ms. However, after a minute test, the mean is 12.47 ± 16.43 ms, although in normal conditions, the microcontroller needs about 7 ms to write all the information within the microSD card. Overall, this test shows that the algorithms implemented require a 2.46% of the microcontroller time among samples. Therefore, the 9 × 3 sensor can be extended to more PD monitoring algorithms such as those presented in Table 5, or other movement analysis techniques related to other fields such as other motor symptoms, well-being or sports.

### 7.3. Algorithm Evaluation

In this section, we report the laboratory evaluation of the two algorithms that detect FoG episodes and bradykinetic gait. From the 12 patients, six suffered FoG episodes in their worst motor state, that is, in the first test when they wore the waist-device without having taken the medication. A total of 106 episodes were recorded and labelled by clinical experts. Each test took between 7 and 10 min and the clinical protocol was repeated twice, since patients paid more attention at their performance on the first test causing a decrease on FoG’s frequency of appearance. It has been shown that clinical environments or new situations can alter the manifestation of FoG episodes [73]. The second time the patient took the test their movements were more natural and more FoG episodes appeared.

Results obtained by comparing the real-time output of the sensor with the video labels show that a total of 87 episodes were detected, leading to a sensitivity of 82.08%. The corresponding specificity was 97.19% according to the episodic evaluation presented in [65].

Regarding the bradykinesia algorithm, since the algorithm is user-dependent, a specific threshold was designed for each patient. The method to set this threshold, which was reported in [66], requires to capture and analyse data previously. Thus, the data collected along 3 days, during which the patient wore the sensor and that are part of Stage 1, was used to acquire the necessary information to personalize bradykinetic gait algorithm. This personalised algorithm was used in real-time in the second part of Stage 1.

Six out of 12 patients presented bradykinesia in the first test (without medication). One of the patients presented bradykinesia at both tests (with and without medication). All the bradykinetic gait episodes were detected, providing a sensitivity of 100%. However, specificity was 93.75% since the algorithm reported a false positive in one of the tests.

Globally, both FoG and bradykinetic gait algorithms achieved good results in the real-time laboratory tests, leading to a good functionality and enable the possibility to activate an auditory stimulus in order to enhance the gait in the following stages of the study.

## 8. Conclusions

PD patients can benefit from an objective evaluation of symptoms, which can be given by means of inertial systems [3,41,60,61]. These devices detect physical measurements that can be evaluated through supervised learning algorithms in order to determine the presence of a symptom. These algorithms are trained based on data obtained from PD patients, therefore, it is interesting to employ an inertial system to both obtain the signals (datalogger functionality) and execute the learning models to test them in the research scope. Furthermore, the usability and comfortability of such devices for the long-term and ambulatory monitoring are crucial and, thus, one single system is considered the most suitable configuration.

To the best of our knowledge, there is not any commercial device able to capture inertial data as well as capable of embedding supervised learning algorithms, as shown in Section 2. Among the commercial inertial measurement units that can be found; none of them meet the requirements mentioned in Section 3. To our knowledge, there is just one system able to capture inertial data and to support the real-time computation of supervised learning algorithms, which is the 9 × 2, previously reported by the authors in [17]. However, this system does not have enough computational resources to compute all the algorithms presented in this paper. Regarding those IMUs able to embed algorithms, they are usually employed for development purposes, being systems without enclosures or without portable energy systems, which makes them unsuitable for ambulatory monitoring. Finally, there is a group of systems employed to only detect PD symptoms. One of the systems is worn on the wrist and the other one is a dual system. However, they cannot detect FoG episodes, neither bradykinetic gait, which is necessary in the MASPARK clinical study to activate RAS.

This paper has presented an inertial measurement unit, called 9 × 3, that is capable of capturing and storing data from different sources: three accelerometers, a gyroscope, a magnetometer and three barometric pressure sensors. Furthermore, the system also enables the real-time computation of supervised machine learning algorithms. The system has been shown to be capable of computing algorithms in real time with accurate results. The system has been employed within the MASPARK project, which aims to enhance gait by means of the correct detection of FoG and bradykinetic gait in real time. In addition to this, given its characteristics, the presented system might be used in other fields such as activity recognition, well-being or sports due to the high data rate and the large full-scale ranges that the system can achieve.

Additionally, the characteristics of the 9 × 3’s microcontroller are much better than the 9 × 2, as reported in Table 3, permitting to include more algorithms and having a much longer autonomy. The device is employed along with a comfortable belt for usability purposes. Furthermore, the sensor is light and small, being suitable for long-term monitoring. As mentioned, the system represents a completely new version of 9 × 2 [17] since it only keeps the enclosure and all the internal components are renewed, including the microcontroller, SD system, sensors, power supply and distribution system. According to Table 1, the system has significantly increased the main features of any commercial inertial measurement unit (e.g., autonomy and sample frequency) and containing all the functional modules (e.g., accelerometers, gyroscope, magnetometer, barometers, the capability of capturing data and embedding algorithms, wireless system, and storage unit). Now, this system enables implementing more complex algorithms due to the enlarged amount of memory and speed that the new microcontroller provides. Furthermore, the triple accelerometer system permits to capture at different ranges of acceleration and enables different functions such as detection of movement or specific orientation. This way, the possibilities given by this device surpass those given by the previous system. Given that high full-scale ranges and sampling frequency up to 1600 Hz are achievable, 9 × 3 is also suitable in other fields such as sports.

The system includes a Bluetooth module in order to synchronize date and time through an external device (such as a smartphone) and a microSD card to store inertial data or the algorithm results. The firmware of the system consists of different blocks managed by a policy of priorities that interact among them. In addition, the system goes to sleep each time it is inactive. As a result, the system can be used in long-term monitoring conditions. According to the measured current, the system has an autonomy of 12.3 days with a 1200 mAh battery satisfying the proposed requirements in Section 3.

The 9 × 3 will be used in the different stages of the MASPARK project by 25 PD patients. First, capturing of inertial signals at home environments takes place during 3 days, which is followed by video-recorded signals at the laboratory. These data will be used to train different supervised algorithms, which will be embedded within the microcontroller, as presented in the results section. The second phase of the clinical protocol will consist of different tests with the aim of testing the algorithms to detect PD symptoms and to eventually activate RAS to improve PD patient’s gait.

This system opens up the possibility of working in several dependency care fields since it has been designed with the ability to capture and store data to be analysed but also the capability of embedding machine learning-based algorithms inside the system in order to use them in real life conditions. The 9 × 3 allows researchers to work with a single system that presents an autonomy of several days working continuously, with different sensors configuration, a very friendly user interface, and with the possibility of sending alarms or messages to external devices as well as to store all the events produced along the data capture.

## Figures and Tables

**Table 1:** List of the main commercial dataloggers.

Name	Manufacturer	Sample Freq.* (Hz)	Autonomy Info.*	Size (mm^3^)	Weight (g)	Storage Unit	Wireless	Acc *	Gyr *	Mag *	Barometric Pressure	GPS
Shimmer 3 [19]	Shimmer	50	450 mAh	51 × 34 × 14	23.6	Yes	Yes	Yes	Yes	Yes	Yes	No
Physilog 4 Gold [20]	Gaitup (EPFL)	500	21 h	50 × 37× 9.2	19	Yes	Yes	Yes	Yes	Yes	Yes	Yes
Physilog 4 Silver [20]	Gaitup (EPFL)	500	21 h	50 × 37 × 9.2	19	Yes	Yes	Yes	Yes	Yes	Yes	No
3-space™ Sensor Datalogger [21]	Yost Labs	475	5 h	35 × 60 × 15	28	Yes	Yes	Yes	Yes	Yes	No	No
MTw Awinda [22]	Xsens	1000	6 h	47 × 30 × 13	16	No	Yes	Yes	Yes	Yes	No	No
MTi-G-710 GNSS [23]	Xsens	375	675–950 mW	57 × 42 × 23.5	55	No	No	Yes	Yes	Yes	Yes	No
KineO [24]	Technoconcept	100	4 h	49 × 38 × 19	25	Yes	No	Yes	Yes	Yes	No	No
Wimu [25]	Realtrack Systems	1000	360 mAh	85 × 48 × 15	60	Yes	Yes	Yes	Yes	Yes	Yes	Yes
3DM-GX4 [26]	MicroStrain	1000	100 mA	36 × 24.4 × 11.1	16.5	No	No	Yes	Yes	No	Yes	No
Dynaport MM [27]	McRoberts	200	14 days	106.6 × 58 × 11.5	55	No	Yes	Yes	Yes	Yes	Yes	No
BioRadio [28]	GLNeuroTech	250	8 h	100 × 60 × 20	113.4	Yes	No	Yes	Yes	No	No	No
Research Tracker 6 [29]	Stayhealthy	20	25 h	51 × 51 × 13	51	Yes	No	Yes	Yes	No	No	No
activPAL^3^ [30]	paltechnologies	10	10 days	53 × 35 × 7	15	No	No	Yes	No	No	No	No
x-IMU [31]	x-IO Technologies	512	150 mA	57 × 38 × 21	49	Yes	Yes	Yes	Yes	Yes	No	No
STT-IWS [32]	STT-Systems	400	3.5 h	56 × 38.5 × 18	46	Yes	Yes	Yes	Yes	Yes	Yes	No
9 × 2 (2013) [17]	UPC-CETpD	200	36.8 h	99 × 53 × 19	78	Yes	Yes	Yes	Yes	Yes	No	No

* Freq, Info, Acc, Gyr, Mag stand for Frequency, Information, Accelerometer, Gyroscope, Magnetometer, respectively.

**Table 2:** Clinical protocol summary.

Stage 0	Stage 1		Stage 2	Stage 3			Stage 4
Baseline exploration	Data capture at patients home	Laboratory validation	RAS personalization	Use system at home without RAS	Washout period	Use system at home with RAS	Use system at home with RAS
Patient’s visit	3 days	Patient’s visit	Patient’s visit	4 days	30 days	4 days	30 days

**Table 3:** Main microcontroller features from the 9 × 2 system and the 9 × 3 system.

Microcontroller Features	dsPIC33FJ128MC804 (9 × 2)	STM32F415RG (9 × 3)
Maximum Speed (Hz)	80	168
Flash Memory (kB)	128	1024
RAM memory (kB)	16	192 + 4 (DMA)
DMA streams	8	16
Consumption at full work (mA) *	65	43
Consumption in Idle mode (mA) *	34	9
Consumption in Sleep mode (mA) *	0.01	0.004
SDIO	No	Yes
I2C Bus	2	3
Computing method	Fixed point	Floating point
Computing performance	40 MIPS	210DMIPS (Dhrystone 2.1)

* Microcontroller’s power consumption in specific work modes: full work is with all peripherals activated and no sleep mode. Idle mode is with the dsPIC working at 80 MHz and the STM32F working at 144 MHz with all peripherals activated. Sleep mode condition works in the dsPIC when only both RTC and external Interrupt are activated and in the case of the STM32F we have the same conditions than dsPIC plus the SRAM Back Up memory activated.

**Table 4:** Feature comparison among the 9 × 3’s barometer sensors.

Parameters	BMP280	LPS25H	MS5637
Range (mbar)	300–1100	260–1260	10–2000
Relative accuracy (mbar)	0.12	0.1	0.1
Absolute accuracy (mbar)	1	1	4
Resolution RMS (mbar)	0.0016	0.000244	0.016
Pressure Noise (mbar)	0.0013	0.01	0.5
Compensation	External	Internal	External
Size (mm^3^)	2 × 2.5 × 0.95	2.5 × 2.5 × 1	3 × 3 × 0.9
Consumption @1 Hz (μA)	2.7	25	20.1
Maximum Data Rate (Hz)	26.7	25	60
Oversampling	16	512	8192

**Table 5:** List of algorithmic blocks performed, the first column shows those calculations needed by the 5 main algorithms listed in the 2nd column. The third column reports the output frequency of each algorithmic block. Note that STFT denotes Short Time Fourier Transform.

Algorithm Block	Algorithm	Temporal Level *
2nd order filters [3,41,60,61,62,63,64]	All	Sample calculation
Mean 3 accelerometer axes [3,41,60,63,64]	Brady, FoG, Gait	Window output
Standard deviation [3,41,60,63,64]	Brady, FoG, Gait	Window output
STFT—Band 1 [3,41,61,62,63,64]	Gait, Dysk, Brady	Window output
STFT—Band 2 [3,41,61,62,63,64]	Gait, Dysk, Brady	Window output
STFT—dyskinetic band [61]	Dyskinesia	Window output
STFT—non-continuous movement band [61]	Dyskinesia	Window output
STFT—Postural Transition band [60,61,63]	Dyskinesia, FoG	Window output
SVM Walk [3,41,61,62,64]	Brady, Dysk, Gait	Window output
Dyskinesia tree-based classifier [61]	Dyskinesia	Window output
Step detector [3,41,62,64]	Brady, Gait	Window output
Stride detector [3,41,62,64]	Brady, Gait	Window output
Cadence Estimation [62]	Gait	Window output
Step length [62]	Gait	Window output
Step velocity [62]	Gait	Window output
Fluidity computation [3,41,62,64]	Bradykinesia	Window output
SVM—FoG yes-no [60,63]	FoG	Window output
Decision tree based classifier for strides [3,41,62,64]	Bradykinesia	Minute output
Dyskinesia 1 min [3,41]	Dyskinesia	Minute output
Bradykinesia 1 min [3,41,64]	Bradykinesia	Minute output
Cadence Estimation 1 min [3,41]	Gait	Minute output
Step length 1 min [3,41]	Gait	Minute output
Step velocity 1 min [3,41]	Gait	Minute output
Tree-based classifier for ON/OFF state [3,41]	ON/OFF	10-min output

* Sample calculation has a frequency of 40 Hz; window output is performed after 3.2 s (1.6 s due to 50% window overlap).

**Table 6:** Patients’ baseline data.

Patients	Gender	H&Y (ON)	H&Y (OFF)	Age	UPDRS III (OFF)	UPDRS III (ON)
Patient 1	Male	2.5	3	62	5	10
Patient 2	Male	2.5	3	69	18	27
Patient 3	Male	2	3	70	7	24
Patient 4	Male	2.5	3	54	21	35
Patient 5	Male	2.5	3	61	8	40
Patient 6	Female	2	3	59	11	20
Patient 7	Male	2.5	3	76	42	45
Patient 8	Female	2.5	3	71	11	24
Patient 9	Female	2.5	3	66	4	12
Patient 10	Male	2.5	3	66	24	35
Patient 11	Male	2	2.5	61	17	32
Patient 12	Female	2.5	3	71	6	17

**Table 7:** 9 × 3 main features.

Name	9 × 3
Manufacturer	UPC-CETpD
Sample Frequency (Hz)	1 to 1600
Autonomy when sampling at 50 Hz	23.09 days at waking hours (Algorithms)
	9.6 days continuously (Algorithms)
	3.81 days continuously (Data Capture)
	9.14 days at waking hours (Data Capture)
Size (mm^3^)	99 × 53 × 19
Weight (g)	83
Storage Unit	Yes
Wireless	Yes
Accelerometer	Yes
Gyroscope	Yes
Magnetometer	Yes
Barometric Pressure	Yes
GPS	No
